# A dynamic approach to unmet needs in long-term care for older people across Europe

**DOI:** 10.1093/geroni/igag042

**Published:** 2026-04-29

**Authors:** Ricardo Rodrigues, Victoria Kontrus, Rebekka Steinlechner, Stefan Hopf, Susana R Marques

**Affiliations:** ISEG Research, ISEG (Lisbon School of Economics & Management), Universidade de Lisboa, Lisboa, Portugal; Vienna Centre for Societal Security (Vicesse), GmbH, Vienna, Austria; Department of Gerontology and Health Research, Karl Landsteiner University, Krems an der Donau, Austria; Vienna Centre for Societal Security (Vicesse), GmbH, Vienna, Austria; ISEG Research, ISEG (Lisbon School of Economics & Management), Universidade de Lisboa, Lisboa, Portugal

**Keywords:** Trajectories, Inequalities, Latent profile analysis, Longitudinal data

## Abstract

**Background and Objectives:**

Studies on unmet needs in long-term care (LTC) have focused on specific time points and have not adopted a dynamic approach that considers changes over time. This study examined the trajectories of unmet needs for care and support with activities of daily living (ADLs) and instrumental ADLs (IADLs) among adults aged 50+ across European countries up to 9 years after the onset of needs.

**Research Design and Methods:**

Using the longitudinal data from the SHARE survey (2011–2022; *n *= 6,154), we used latent profile analysis to identify 5 trajectory types defined by time until onset of unmet needs, number of changes of state, time spent with unmet needs, ADL/IADL limitations, and age at onset of needs. These trajectories are marked by early periods of unmet needs (short-delayed met needs; long-delayed met needs; high needs and delayed), ambivalent (U-shaped met needs), or outright positive (met needs from onset).

**Results:**

An overwhelming majority of older adults experienced unmet needs at some point after the onset of needs. Multivariate analysis shows that having a cohabiting spouse/partner and higher income are associated with trajectories where care is available early on. Country-level public expenditure on LTC is also a strong determinant of belonging to different trajectories. We further explored the association between these trajectories and mortality and ADL/IADL limitations.

**Discussion and Implications:**

Results confirm the relevance of transition points, such as the time around the onset of needs, highlighting the potential role for case management and discharge teams at such transitions.

Innovation and Translational Significance: Using longitudinal data and latent profile analysis, this is the first study to conduct a comparative analysis of time trajectories of unmet needs for care and support with ADLs/IADLs and their determinants across long-term care systems in Europe. Unmet needs were observed mostly immediately after the onset of needs, highlighting the need for case management or discharge teams, but spells of unmet needs continue or are also observed in later periods. The prevalence of unmet needs long after the onset of needs underscores the need for periodic assessments and screening of frail older people.

## Background and theoretical frameworks

Longer life expectancy, as well as an increasing share of older people with chronic conditions and multimorbidity, has increased the demand for care and support to older people across Europe (European Commission, 2021). In response to this, there has been a growing investment in home care services as a way to supplement informal care (unpaid support provided by family members, friends, or neighbors), and to allow older people to age in place (European Commission, 2021). Despite this investment, concerns remain regarding the ability of long-term care systems to meet this growing demand for care, particularly as family structures change and informal care may not be as readily available as in the past (Rodrigues et al., 2023). In an apparent confirmation of these concerns, there is evidence of pervasive unmet needs for care and support with activities of daily living (ADLs) and instrumental ADLs (IADLs) across Europe and the United States (Calderon-Jaramillo & Zueras, 2023; LaPlante et al., 2004). This in turn may lead to higher mortality risk, deteriorating health and quality of life, and greater risk of loneliness (He et al., 2015; Huang et al., 2022; Wang et al., 2024).

Unmet needs arise when a person has limitations but does not receive support (Allin & Masseria, 2009). The literature has used two distinct definitions of unmet need: (a) *subjective unmet needs*, based on the self-reported assessment of the adequacy of care, and (b) *objective* or also called *absolute unmet needs*, based on whether or not respondents receive care for an activity for which they report having limitations (Spiers et al., 2022). While subjective unmet needs may be influenced by expectations of what constitutes “met needs” and therefore are less suited for comparative research (Allin & Masseria, 2009), objective measures of unmet needs identify those most in need of care and thus have greater policy relevance (Spiers et al., 2022). In this study, we focus on objective unmet needs.

A common framework used to analyze unmet needs is Andersen’s (1995) Behavioral Model of Care. This framework models the use of care as depending on system or environmental factors (e.g., generosity of the long-term care system) and population characteristics. The latter are divided between predisposing factors (e.g., age, gender), enabling factors (e.g., income, size, and composition of the household or family), and needs (e.g., limitations with ADLs and IADLs). Andersen’s (1995) Behavioral Model of Care Use provides a useful framework for understanding how the mismatch between needs and use of care may vary across population groups. Available evidence covering very different care contexts and long-term care systems confirms that unmet needs are concentrated on people with lower social status or those living alone (Calderon-Jaramillo & Zueras, 2023; Chou et al., 2024; Szenkurök et al., 2025). Conversely, the risk of experiencing unmet needs for care and support with ADLs and IADLs seems to be lower among the oldest old, unhealthier individuals and women, indicating the efficacy of targeting mechanisms toward more severe cases in long-term care systems in Europe (Calderon-Jaramillo & Zueras, 2023; Szenkurök et al., 2025). The same studies, however, reach dissimilar conclusions as to whether differences in long-term care systems’ generosity or types of benefits provided—the environmental factors depicted in Andersen’s model—are associated with differences in unmet need.

Despite the wealth of studies on unmet needs for care and support with ADLs and IADLs (e.g., Kalánková et al., 2021; Spiers et al., 2022), the majority of this literature has taken a static approach to unmet needs. However, Smith and Connolly (2020) argue that the absence of a dynamic perspective conceals the heterogeneity of unmet needs as it fails to consider the *duration* of unmet needs and to distinguish needs that are *never translated into demands* (e.g., because they resolve themselves in a short period of time without need for care), needs that are *delayed* in their resolution or *never met at all*. In the absence of a specific theoretical framework for taking a dynamic perspective on unmet needs (Smith & Connolly, 2020), we propose to draw on life course theories to conceptualize trajectories of unmet needs, specifically using the concepts of timing and sequence of unmet needs and cumulative processes.

Life course theories depict life as a sequence of episodes defined by key life events (e.g., entering the labor market, widowhood) and transitions in the trajectories of individuals (Elder et al., 2003). These transitions are often defined around age or normative categories (e.g., childhood, old age), but they may also be defined around key turning points (Lawless et al., 2026). While several authors have recently begun to recognize caregiving as a separate domain or life trajectory (Keating et al., 2019; Lawless et al., 2026), we propose to extend this by using the onset of needs as a key turning point in trajectories of unmet need. A significant concept in the life course literature, *pathways* or *trajectories* are defined around sequences of events or transitions that mark the bookends of life stages (Lawless et al., 2026). Adapting this to unmet needs, trajectories of unmet need can be defined by the sequence or patterns of met and unmet needs that are observed once older people first experience the onset of needs. Consequently, it is relevant to distinguish between a succession of short unmet needs episodes—similar to what Fast et al. (2019) coined as “serial care” in relation to caregiving episodes—from a single long spell of unmet needs, for example.

Besides the duration and sequence of periods of met and unmet need, another key concept, as pointed out by models focusing on the accumulation of risks (Ben-Shlomo & Kuh, 2002), is *timing* or when such transitions occur. For example, failure to have needs met immediately after the onset of frailty may trigger a process of cumulative disadvantage that leads to longer spells of unmet needs or a quicker deterioration of someone’s condition, therefore exacerbating needs. Similarly, the point in one’s (older) life stage when the onset of needs occurs may also be important to understand these cumulative processes and subsequent trajectories. For example, the onset of needs at a later age, when widowhood is more likely, may negatively influence the possibility of having needs met (Hu et al., 2022; Ilinca et al., 2022). Finally, the severity of needs at the onset of the unmet needs trajectories—in itself a possible result of accumulated disadvantages over the life course—may also place individuals on different subsequent pathways.

Using life course concepts to study trajectories of unmet needs also proves useful to analyze another of Smith and Connolly’s (2020) propositions: the possibility of heterogeneous patterns of unmet needs, for example, as to the duration or sequence of unmet needs across time. Some studies on trajectories of unmet needs for care and support with ADLs and IADLs in old-age that have indeed hinted at the diversity of such trajectories. For example, Wang and colleagues (2024) described “increasing,” “persistent,” and “decreasing” trajectories of unmet needs among Chinese older people, with the increasing trajectory associated with older age groups, ethnic minorities, and socio-economic disadvantages. Freedman and colleagues (2024) find an association between trajectories of increasing needs in the United States and a higher probability of unmet needs. However, most of these and similar studies on trajectories of unmet needs have relied on relatively short follow-up periods and thus have been unable to examine long-term trajectories of unmet needs or focus on a single country (Cloutier et al., 2019; Gonçalves et al., 2023; Hu et al., 2022). To our knowledge, this is the first study to analyze trajectories of unmet needs across different types of long-term care systems, spanning up to 9 years of data and specifically addressing the heterogeneity of trajectories of unmet needs.

This study aims to take a dynamic perspective on unmet needs at the individual level by analyzing how they evolve since the onset of needs. Two complementary theoretical perspectives inform our main research questions. Informed by the concepts of life course theories described above, the first question is whether there are heterogeneous patterns of unmet needs over time, defined by differentiated trajectories of unmet needs. Building on Andersen’s Behavioral Model, the second question is whether trajectories with worse profiles of unmet needs (e.g., longer consecutive or interspersed periods of unmet needs) are associated with more vulnerable groups or characteristics.

## Research design and methods

### Data source and sample

This study uses data from the Survey on Health, Ageing and Retirement in Europe (SHARE), specifically waves 4 to 9, collected between 2011 (2010 for Estonia) and 2022 (SHARE-ERIC, 2024a, 2024b, 2024c, 2024d, 2024e, 2024f). SHARE is a cross-national longitudinal survey representative of the population aged 50 and older residing in the community in several European countries (Börsch-Supan et al., 2013). Waves 5 to 9 were selected as the more recent SHARE waves with information on the use of both home care services and informal care, while Wave 4, despite not having information on the use of home care services, contains information on whether interviewees have needs or not. Home care refers to personal care, help with domestic tasks, or meals on wheels received from professional services in one’s own home, while informal care refers to personal care received from a co-residing household member.

Consecutive selections of observations were carried out to assemble trajectories of unmet needs for care and support with ADLs and IADLs in old age (see Supplementary Figure 1 for the sample selection process). The total initial sample of 139,923 individuals who are uniquely identified in at least one wave after wave 4 was first restricted to those fulfilling two additional criteria: (a) when first sampled into SHARE in Wave 4 or later have no self-reported limitations with ADLs nor IADLs, and no self-reported cognitive impairment (the latter defined as being diagnosed or currently having Alzheimer’s disease, dementia or senility)—henceforth referred to as “needs”—and (b) who subsequently go on to report needs in at least one wave. A further restriction criterion was not having data gaps of two or more waves after the onset of needs, as allowing for such gaps would, in practice, entail a period of a minimum of 4 years for which no information would be available. Finally, we also excluded individuals for whom the total number of waves after the onset of needs was only two or for whom data were missing for any of the variables used in the study. The final sample therefore consists of an unbalanced panel of 6,154 individuals, each corresponding to one trajectory, comprising in total 21,806 time observations (see Tables 1 and 2 for the descriptive statistics of the final sample). During the study period, 637 trajectories were truncated due to the death of the individual (10.4% of the sample of individuals/ trajectories). The number of missing time observations due to individuals dropping out of the sample is 2,435 (9.7% of all observations should the panel have been balanced, and five data points would have been observed for each individual) (see Supplementary Figure 2 for a graphic representation of the different states across waves).

**Table 1:** Mean values of core characteristics by trajectory of unmet needs.

Variables	U-shaped met needs	High needs and delayed met needs	Met needs from the onset	Long-delayed met needs	Short-delayed met needs
Time (waves) until first unmet need	1.60	0.44	4.99	0.07	0.01
Duration of unmet need (%)	31.89	45.56	0.20	73.85	33.06
No. of changes between states	2.19	1.78	1.47	1.17	1.73
Age onset of needs	74.88	74.17	75.28	72.97	71.84
No. of ADLs at onset	0.76	4.51	0.72	0.54	0.46
No. of IADLs at onset	1.54	6.55	1.57	1.11	0.95
Share of total sample (%)	12.56	1.66	23.16	19.66	42.97

*N *= 6,154. ADL, activities of daily living; IADL, instrumental activities of daily living.

**Table 2:** Bivariate analysis of determinants of trajectories of unmet needs.

Variables	U-shaped met needs	High needs and delayed met needs	Met needs from the onset	Long-delayed met needs	Short-delayed met needs	*p*
**Sex**						
Female	44.9	39.1	34.7	39.6	43.7	.057
Male	55.1	60.9	65.3	60.4	56.3	
**Partner**						
Partner in HH	49.6	67.9	42.3	59.2	65.2	<.001
No partner in HH	50.4	32.1	57.7	40.8	34.8	
**Adult child**						
Adult child in HH	16.8	32.4	16.2	19.2	21.4	.081
No adult child in HH	83.2	67.6	83.8	80.8	78.6	
**Income**						
First quartile	38.1	39.7	34.4	38.8	39.5	.167
Second quartile	28.2	33.8	22.4	22.2	21.5	
Third quartile	19.3	13.2	19.7	19.8	16.4	
Fourth quartile	14.3	13.3	23.5	19.2	22.6	
**Education**						
Primary	37.5	47.6	33.5	50.1	33.6	.002
Secondary	46.0	41.8	49.9	41.1	51.9	
Tertiary	16.5	10.6	16.6	8.9	14.6	
**Country cluster**						
Low expenditure	2.0	2.7	2.9	8.4	3.3	<.001
Mid-low expenditure	23.4	32.9	17.6	26.9	25.6	
Mid-high expenditure	71.1	64.0	74.1	60.8	66.4	
High expenditure	3.6	0.5	5.4	4.0	4.7	
**Change in income**						
No change or improvement	43.8	42.9	48.4	47.1	42.7	.425
Decrease	56.2	57.1	51.6	52.9	57.3	
**Change in HH size**						
Same size or larger	80.0	76.7	81.0	84.0	80.7	.714
Smaller	20.0	23.4	19.1	16.0	19.3	

*N *= 6,154. HH, household. Values represent percentage. Weighted results (calibrated cross-sectional individual weights for the first wave in which each individual joined the sample).

### Measurements

Unmet needs for care and support with ADLs and IADLs were assessed in an absolute manner through a binary variable coded as having unmet needs in a given wave if individuals did not report receiving any help or support from home care services (henceforth “formal services”), or informal carers, whenever needs were present in a given wave. In each wave, the variable was coded as having no unmet needs if individuals reported needs and receiving any help or support in that wave, or if they reported no needs in that wave after the onset of needs (this latter case could stand for what could be initial shorter periods of dependency that were then reversed (Smith & Connolly, 2020). For example, an individual may have first reported needs in wave 5, without receiving care in that wave, and subsequently reported no needs in wave 6, in which case this individual would have been coded as having unmet needs in wave 5 and no unmet needs in wave 6. Objective unmet needs minimize a known issue with subjective unmet needs: as those reporting subjective unmet needs tend to use care more often than those who do not report unmet needs (even after controlling for health), subjective unmet needs may capture dissatisfaction with care received rather than actual forgone care or barriers to the use of care (Allin et al., 2010). Such over-estimation is unlikely to take place with the definition of unmet needs used in the present study.

For the identification of trajectories of unmet needs, six variables were used. The choice of these variables was informed by the life course concepts discussed above, particularly what Keating and colleagues (2019) defined as key elements of trajectories of care used in life-course analyses of caregiving (Fast et al., 2020) and adapts them to unmet needs. These variables include the transition into and out of unmet needs (*bookends* of trajectories), episodes, and the sequence of unmet needs. Therefore, we used the time until onset of unmet needs, measured as the number of waves until unmet needs were first coded once needs arose, and total time spent with unmet needs, measured in percentage of the total number of waves observed in each trajectory. We also included the number of changes of state from met to unmet needs or vice versa in the trajectory. To further characterize these trajectories, three additional variables measured at the onset of needs were also included: the number of ADL limitations, the number of IADL limitations, and age. These capture the severity of needs at the start of the trajectories that may result from cumulative processes, as well as the timing of transitions along the life course. These three variables were continuous. Although included in the original definition of needs above, cognitive impairment was not used in identifying trajectories due to high collinearity with ADL limitations.

Following the existing literature on unmet needs and Andersen’s Behavior Model of Care described above, a number of covariates were used to analyze the determinants of belonging to each trajectory. These include sex, existence of a cohabitating partner or spouse, and existence of a cohabitating adult child, all measured as binary variables. As for socio-economic variables, we included the respondent’s quartile of equivalized household income (total household income divided by the square root of the household size) calculated at the country and wave level, and the highest educational achievement (primary, secondary, and tertiary). Possible deteriorating access to material or household resources (for example, to aid in accessing care), was assessed through two binary variables on deterioration or reduction of income (as opposed to maintaining or increasing income—assessed as a change in equivalized income adjusted for inflation, instead of change of income quartile so as to have sufficient variation) and reduction of household size (as opposed to maintaining or increasing household size), in both cases comparing the last observation with the one at the onset of care needs. To capture institutional or demand-side context, we included also a country-level variable that classified countries according to their level of public expenditure on care long-term care (European Commission & Pavolini, 2022): low (equal or below 0.6% of GDP), medium–low (between 0.7% and 1.3%), medium–high (between 1.5% and 2.2%), and high public expenditure (above 2.3% of GDP). This typology was originally developed using data for 2019 (roughly a midway point in the data used in this study). Variations in public expenditure on long-term care between 2013 and 2022 were assessed using data from successive Ageing Reports (cf. EPC-AWG/EC, 2024), which confirm that these variations were minimal and did not affect this typology.

### Statistical analysis

We first used latent profile analysis (LPA) to identify trajectories of unmet need. As with other finite mixture models, LPA enables the identification of subgroups and is therefore suitable to assess the heterogeneity within a given sample using continuous or a mixture of continuous and categorical variables (Dyer & Day, 2015; Nylund et al., 2007). The LPA was estimated using the full information maximum likelihood estimator (Masyn, 2013). Each subgroup or cluster–in the case of the present study, each trajectory—is considered to be a latent variable or cluster, with group membership and comparisons taking this probabilistic characteristic into consideration. To identify the trajectories of unmet needs, the six above-described variables are included in the model, and a decision on the number of clusters or trajectories was taken using a combination of indices to assess statistical fit. Specifically, for each model with a different number of trajectories, we estimated the Bayesian Information Criterion (BIC), the Akaike Information Criterion (AIC), the log-likelihood, the Vuong, Lo-Mendell, Rubin (VLMR) likelihood test, entropy, the minimum number of cases in the smallest cluster in each trajectory, and average posterior probabilities associated with each trajectory (Nylund et al., 2007; Stanley et al., 2017). While some literature reports superior statistical power for the bootstrapped Lo-Mendell, Rubin test, particularly for smaller samples (Nylund et al., 2007), some reservations have been expressed as to its use with complex survey data or its propensity to favor larger cluster models (Sinha et al., 2021; Weller et al., 2020). Given the large sample size of the present study and as the results below supported either a four- or five-trajectory model, we opted to include the VLMR test instead as a more conservative approach. Moreover, existing evidence has shown that with samples larger than 1,000, the above-mentioned Information Criteria correctly identified the models in the data (cf. Sinha et al., 2021).

Subsequently, each of the resulting trajectories was then analyzed to confirm if they were clearly differentiated from each other. To this end, we compared trajectory membership in relation to each of the six core variables used to define the trajectories, as well as a number of additional covariates, using one-way ANOVA tests. This bivariate analysis of the characteristics of each trajectory employed SHARE’s calibrated cross-sectional individual weights for the first wave in which each individual joined the sample. A bivariate analysis of trajectory membership and the six core variables, as well as the multinominal logistical regression for the determinants of trajectory membership described in the next paragraph were also re-estimated using weights. Results remained the same (see Supplementary Tables 1 and 2).

Finally, following Vermunt’s (2017) approach to estimate determinants of trajectory membership that accounts for the probabilistic nature of class membership, the posterior class probabilities of belonging to each trajectory produced by the LPA were used to assign each individual to a trajectory. We then used this trajectory membership as the dependent variable in the estimation of a multinominal logistical regression for the determinants of trajectory membership. Given the sample sizes, we considered statistical significance at *p *< .05. All analyses were performed using STATA version 18 and the user-written program *STEP3* (Califano, 2023).

## Results

### LPA analysis on the number of trajectories

Table 3 presents the results for the different indices and measures of fit across models with a number of trajectories ranging from 1 to 6. The main measures of fit and substantive theoretical analysis of the resulting trajectories supported both a four- and five-trajectory model. After comparing the characteristics of each of the trajectories in these two models, we opted for the one with five trajectories as the BIC values clearly levelled only after that model, and the additional smallest trajectory in this model had a clear research and policy relevance, enabling a more nuanced understanding of trajectories of unmet needs, as shown below. While the literature urges caution when the smaller cluster size is reduced, which in this case corresponds to only 1.66% of the total sample, such a recommendation is based on studies employing much smaller sample sizes than the one employed here. Results for the four-trajectory model were not significantly different, except for the absence of the smallest trajectory, which was distributed among the other four (results available from the authors on request). Entropy levels were all very high, clearly showing that trajectories were sufficiently differentiated.

**Table 3:** Fit indices for latent profile class models.

Number of classes	AIC	BIC	Log-likelihood	SABIC	VLMR (*p*-value)	ALCPP	Entropy	Smallest %	Smallest *N*
1	181,428	181,508.7	−90,702.02	181,476.2	–	–	–	100	6,154
2	167,303.2	167,431	−83,632.61	167,409.4	.000	0.9998	0.999	24.28	1,494
3	161,786.2	161,961.1	−80,867.11	161,950.6	.000	0.9996	0.997	23.16	1,425
4	157,464.3	157,686.2	−78,699.15	157,686.8	.000	0.9901	0.980	13.18	811
5	155,413.7	155,682.7	−77,666.85	155,694.3	.000	0.9806	0.978	1.66	102
6	154,987.7	155,303.8	−77,446.84	155,326.4	.000	0.8115	0.948	1.60	98

*Note. N *= 6,154. AIC = Akaike Information Criterion; ALCPP = Average Latent Class Posterior Probability; BIC = Bayesian Information Criterion; SABIC = sample-adjusted Bayesian Information Criterion; VLMR = Vuong–Lo–Mendell–Rubin likelihood ratio test.

**Figure 1:**
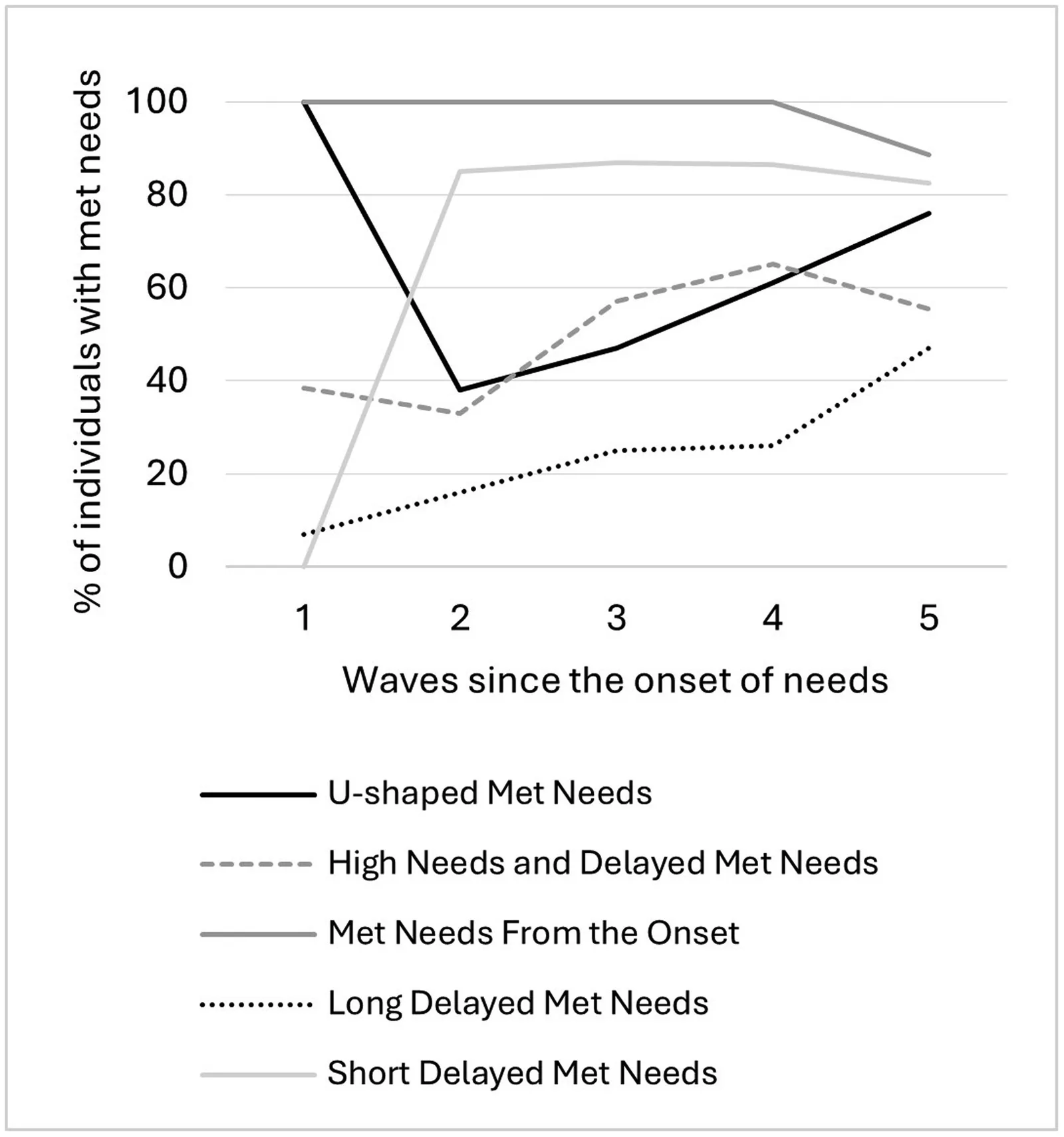
Trajectory membership and prevalence of met and unmet needs across time. *N* = 6,154.

The five trajectories were labelled according to their main components (Table 1), as well as Smith and Connolly’s (2020) typology of dynamics of unmet needs. The trajectory comprising the largest number of individuals (42.97%) was the *short-delayed met needs*. This trajectory was foremost characterized by a spell of unmet needs immediately at the onset of needs, which was met in the subsequent wave, in which individuals were observed and may have been repeated subsequently, since the mean number of changes of state was close to 2. After the initial period, the percentage of individuals with unmet needs in this trajectory remained low (Figure 1). This is also a trajectory defined by the lowest age and best functioning status (measured by the mean number of ADL and IADL limitations) at the onset of needs of all trajectories. The next most frequent trajectory was *met needs from the onset* (23.16%), which had the most favorable profile. Overall duration of unmet needs was residual, and if at all observed, it was at the end of the trajectory. Individuals in this trajectory were the oldest on average across the different trajectories but had good overall functional status at the onset of needs. The *long-delayed met needs* trajectory represented 19.66% of the sample, and, similar to the *short-delayed met needs* trajectory, was defined by unmet needs immediately at the start of the trajectory, which, in this case, lasted for the majority of the time during which individuals were observed in the sample, until needs were eventually met. With the exception of the period at the onset of needs, the long-delayed met needs showed the lowest share of adults reporting met needs over time among all trajectories (Figure 1). Although a year older on average at the onset of needs than those in the short-delayed met needs, individuals in the long-delayed trajectory were not significantly worse off in terms of functioning status at the onset of needs. In fact, they had the lowest mean number of ADL and IADL limitations after those in the short-delayed trajectory. The *U-shaped met need* trajectory (12.56%) was thus named as its characteristics suggest a trajectory in which needs are initially met, only to be left unmet halfway through and again met at the end of the time during which individuals are observed. The age and limitations profile at the onset of needs is very similar to that of the Met Needs from the Onset above, suggesting other factors in play for the development of this trajectory. Finally, the smallest cluster or trajectory is the *high needs and delayed met needs*, which accounts for only 1.66% of the total sample. As the moniker indicates, there is an initial spell of unmet needs that was eventually met. In this respect, it was similar to the short-delayed met needs trajectory (also in experiencing unmet needs again later in the trajectory), although its average overall duration of unmet needs was somewhat longer, and the mean number of limitations with both ADLs and IADLs at the onset of needs was much higher than those observed for any of the other trajectories.

### Determinants of care trajectories

Having used the six variables depicted in Table 1 to define each trajectory, we then used the posterior class probabilities of belonging to each trajectory as a dependent variable to determine correlations with other socio-economic variables through bivariate and multivariate statistical analysis. When comparing the five trajectories, there were only statistically significant differences among the five for the existence of a partner in the household, education level, and the country-level variable (Table 2). Cohabitating with a partner was associated with the high needs and delayed met needs, long-delayed and short-delayed met needs trajectories, which are the trajectories in which individuals reported greater difficulties in meeting needs when these first arose. Regarding education level, there seems to be a gradient in the different trajectories of unmet needs. Primary education is the mode for the high needs and delayed met needs and long-delayed met needs trajectories, while those with tertiary education are over-represented in the u-shaped met needs, met needs from the onset and short-delayed met needs trajectories. At the country level, the variable depicted in Table 2 represents the available resources in long-term care in relative terms across Europe. Low public expenditure countries are over-represented among the long-delayed met needs trajectories, while individuals from high public expenditure countries are hardly found in the high needs and delayed met needs trajectory. Mid-high public expenditure countries are over-represented among the met needs from the onset and U-shaped met need trajectories, while mid-low public expenditure countries are more prevalent in the high needs and delayed met needs trajectory.

The multivariate analysis of the determinants of belonging to the different trajectories only partially confirms the results of the bivariate analysis (Table 4), thus showing the existence of composition effects in the determinants of trajectory membership. The main determinants of belonging to the U-shaped met needs trajectory, assessed at the onset of needs, were having a cohabitating partner (although no association was reported for cohabitating adult children) and belonging to the second- and third-income quartile, while the most affluent individuals were not more likely to belong to this trajectory than those in the reference category (the first-income quartile). At the country level, higher public expenditure on long-term care reduced the probability of belonging to this trajectory, as did reductions in household income after the onset of needs. Most determinants of the high needs and delayed met needs trajectory were not statistically significant (at the *p < *.05 level at least)—likely due to the low sample size—with the exception of high public expenditure countries, which were negatively associated with this trajectory. As for the met needs from the onset trajectory, being a woman, belonging to a higher-income quartile, or residing in a country with a higher public expenditure on long-term care were all positively associated with this trajectory. As for household size and composition, their association was differentiated. Having a cohabitating partner at the onset of needs and reporting a reduction in household size at the end of the observation period increased the probability of belonging to this trajectory, while having cohabitating adult children showed the opposite association. The long-delayed met needs trajectory had nearly the exact opposite determinants of the care from the onset trajectory. the exception was education levels, in which higher formal education was negatively associated with this trajectory, while there was no such association with met needs from the onset. Finally, those associated with the short-delayed met needs were individuals not cohabitating with their partner or spouse, with higher formal education, and residing in either countries with high or mid-low public expenditure on long-term care. Individuals in higher income quartiles were less likely to belong to this trajectory, while reporting a reduction in income between the onset of needs and the last observation in the sample was positively correlated with this trajectory.

**Table 4:** Multivariate analysis of determinants of trajectories of unmet needs.

Variables	U-shaped met needs		High needs and delayed met needs		Met needs from the onset		Long-delayed met needs		Short-delayed met needs	
	AME	*p*	AME	*p*	AME	*p*	AME	*p*	AME	*p*
Female (ref: male)	−0.009		−0.006	¥	0.041	***	−0.022	*	−0.003	
Partner in HH (ref: no partner in HH)	0.061	***	−0.004		0.157	***	−0.058	***	−0.155	***
Adult child in HH (ref: no adult child in HH)	−0.019		0.006		−0.053	**	0.038	*	0.028	
**Income (ref: 1st quartile—poor)**										
Second quartile	0.057	***	0.001		0.072	***	−0.056	***	−0.074	***
Third quartile	0.037	**	−0.001		0.099	***	−0.057	***	−0.079	***
Fourth quartile	−0.006		−0.001		0.120	***	−0.074	***	−0.038	¥
**Education (ref: primary)**										
Secondary	0.003		−0.002		−0.019		−0.046	***	0.065	***
Tertiary	−0.002		−0.004		0.021		−0.067	***	0.053	**
**Country cluster (ref: low expenditure)**										
Mid-low expenditure	−0.034	*	0.005		0.046	**	−0.067	***	0.050	*
Mid-high expenditure	−0.029	*	−0.008	¥	0.144	***	−0.107	***	0.000	
High expenditure	−0.076	***	−0.018	***	0.147	***	−0.104	***	0.050	*
Reduction in income (ref: no change or improvement)	−0.036	***	0.004		−0.017		−0.005		0.054	***
Reduction in HH size (ref: same size or larger)	0.009		−0.002		0.059	***	−0.049	***	−0.016	

*Note. N *= 6,154. AME = average marginal effect; HH = household; Ref = reference.

¥ *p *< .1. *p < .05. **p < .01. ***p < .001.

### Sensitivity analysis

As mentioned before, 10.4% of individuals in the final sample have their trajectories right-censored due to death. This raises the question of whether such an outcome is systematically associated with any of the trajectories, such as those with more adverse profiles of unmet needs, such as high needs and delayed met needs or long-delayed met needs. The share of trajectories that are censored due to death is indeed higher in the former trajectory (Table 5), but only marginally, and it is nearly as high as that in the met needs from the onset trajectory. Although differences in the share of individuals who passed away across trajectories are statistically significant overall, this is mostly driven by the much lower prevalence of censoring due to death among the short-delayed met needs trajectory.

**Table 5:** Bivariate analysis of the association of death with trajectories of unmet needs.

Variables	U-shaped met needs	High needs and delayed met needs	Met needs from the onset	Long-delayed met needs	Short-delayed met needs	*p*
Deceased during sampling	12.2	14.3	13.2	10.9	6.2	.000
Alive throughout	87.8	85.7	86.8	89.1	93.8	

*Note. N *= 6,154. Weighted results (calibrated cross-sectional individual weights for the first wave in which each individual joined the sample). Values represent percentage.

In parallel, we have also compared the evolution of the mean number of limitations with ADLs and IADLs across different trajectories and over time after the onset of needs (given our sample selection criteria, there are no limitations with ADLs or IADLs before wave 1; see Supplementary Figure 3). For most trajectories, the mean number of limitations with ADLs is relatively flat (Supplementary Figure 1, Panel A). There is only a limited deterioration in the mean limitations with ADLs across time for the long-delayed met needs and the U-shaped met needs trajectories, which in the latter trajectory occurs during the period of unmet needs. There is no deterioration at all for the short-delayed met needs and met needs from the onset trajectories. The most visible difference is observed for the high needs and delayed met needs, whose individuals report a very high mean value for the number of limitations with ADLs at the onset of needs, which then drops steadily afterward. For the limitation with IADLs, the picture is very similar (Supplementary Figure 1, Panel B), with arguably larger differences between trajectories in the mean values and more pronounced deterioration of needs for the long-delayed care and U-shaped met needs. As with ADL limitations, the high needs and delayed met needs trajectory shows an overall reduction in the mean number of limitations with IADLs.

## Discussion and implications

Results clearly confirmed Smith and Connolly’s (2020) proposition that unmet needs have a dynamic and heterogeneous nature across time (Smith & Connolly, 2020). Relying on concepts derived from life course theories, such as timing, duration, and trajectories or pathways, five trajectories related to unmet needs were identified in our sample. In at least two of these—long-delayed met needs and high needs and delayed met needs—failure to meet needs at their onset placed individuals on longer trajectories of unmet needs. While representing a reduced sample, the high needs and delayed met needs group was also present in other longitudinal studies of needs and care use (Hu et al., 2022). This identified the onset of needs as a critical period in trajectories of unmet need. This could also imply the relevance of timing of unmet needs in generating or building on cumulative processes of disadvantage (Ben-Shlomo & Kuh, 2002), particularly for those in the high needs and delayed met needs trajectory, which had the worst profile of needs at the onset of the trajectory. However, this was somewhat contradicted by those in the short-delayed unmet needs category, who also experienced an initial spell of unmet needs from which they quickly rebounded. This trajectory seems to come closer to what Smith and Connolly (2020) termed “no use of care” due to short-term conditions that resolve themselves without the need for care or to the adaptation of individuals to low-level needs through self-care, as individuals in this trajectory reported the best overall health condition at the onset of needs. In this sense, it is closely aligned with the “Low then resolved” trajectory of needs identified among U.S. Medicare users (Freedman et al., 2022) and the “Decreasing” unmet needs trajectory in Wang et al.’s study (2023). This trajectory could also refer to instances where access to formal services was only possible after the reduction in income met the means test in mid-low public expenditure countries. Experiencing the onset of needs at different stages in the (old age) life course was associated with different trajectories, but older individuals were not necessarily associated with more adverse trajectories. Life-course concepts proved valid to underpin trajectories of unmet needs, and these may need to be considered as relevant life events in older stages of the life course.

We next used Andersen’s (1995) Behavior Model of Care to underpin the analysis of the determinants of trajectories of unmet needs. Previous research had shown that household size and composition were both positively associated with a lower probability of reporting unmet needs (Hu et al., 2022; Szenkurök et al., 2025). In our results, having a cohabitating partner or an adult child at the onset of care needs has different implications for the trajectories of unmet needs. The former is clearly associated with trajectories in which care is available at the onset of needs (U-shaped met need and met needs from the onset), either because spouses and cohabitating partners can step in to provide care themselves, or they can advocate for or help those in need of care navigate complex care systems. This confirms the findings from previous studies on unmet needs (e.g., Hu et al., 2022; Szenkurök et al., 2025). The presence of adult children in the household at the time of the onset of needs is associated with the long-delayed met needs trajectory, indicating that some forms of intergenerational cohabitation may not be primarily driven by provision of upwards intergenerational care (Dykstra, 2021). Larger household sizes at the end of the trajectory were nonetheless associated with this trajectory, which could mean needs were nonetheless eventually met by relatives or acquaintances who moved in with the person in need of care. Higher income allowed early access to care at the moment that needs started, while the opposite was associated with delayed care trajectories, which seems to corroborate the findings of other studies that included a dynamic perspective of unmet needs (Hu et al., 2022; Wang et al., 2024). Secondary and tertiary education were each associated with the short-delayed met needs—likely denoting individuals with higher health literacy who could manage their condition without recourse to care—while those with primary education were more likely to experience long-delayed met needs. The country context, specifically the level of public resources devoted to long-term care, which can be proxied as the availability of formal services or supported familialism through cash-for-care, was arguably the most relevant determinant of trajectories of unmet need, which shows the relevance of public policies in supporting access to care. Results provide a more nuanced picture of determinants of unmet needs, which calls for the incorporation of dynamic aspects into Andersen’s Behavioral Model of Care.

One major concern around unmet needs for care and support with ADLs and IADLs is that they may lead to a (quicker) deterioration of needs with time (He et al., 2015; Huang et al., 2022). Establishing causality between the different trajectories and the evolution of care needs was beyond the scope of this study. Nonetheless, results did show that trajectories associated with longer delays in having needs met or U-shaped trajectories of met needs seem to be associated with increased needs. The high mean number of both ADLs and IADL limitations observed among individuals in the high needs delayed met needs trajectory immediately at the onset of needs is compatible with the aftermath of an acute health event (Gonçalves et al., 2023), and although there was a recuperation to the mean number of limitations observed among the other trajectories, this only occurred after some time. Uncovering causal links between the timing and duration of unmet needs for care and support with ADLs and IADLs and the evolution of limitations and health conditions in general is a further avenue of research.

### Limitations

The definition of unmet needs used in this study is an objective and absolute definition (the only available in SHARE) that only considers use and not intensity of care or a disaggregation of needs. The effect of this definition, as opposed to using self-reported unmet needs are difficult to ascertain. While this may underestimate unmet needs by not accounting for care that is present but insufficient to fully meet needs (although it is not clear how this could be distributed across the different trajectories), some cases classified as unmet needs may not be considered as such by individuals themselves (Gibson & Claire, 2019). This might be the case with some of those in the short-delayed met needs trajectory. Both intensity and disaggregation of care needs remain worthwhile possibilities for further research. Still on the definition of met and unmet needs, for analytical tractability, met needs are not distinguished from periods of no need (after the first onset of needs at least) in this study. This risk conflating two different states, which should merit further research in the future. The sample size for the trajectory High Needs and Delayed Met Needs may have precluded real associations with some determinants from being uncovered or raised issues of generalizability, and results for this trajectory should be interpreted with caution. The trajectories depicted here have a long follow-up period, allowing for the exploration of longer-term variation in need and unmet need. The downside of this longer follow-up period is attrition. The number of missing time observations is just under 10%, and those that are truncated due to death are also limited in number and seemingly uncorrelated with any of the trajectories, but one cannot really fully correct for this attrition.

### Policy implications

The results show that the period around the onset of needs is critical for experiencing unmet needs, affecting the majority of individuals in our sample. This highlights the importance of managing transitions around that period, which can be accomplished by discharge teams in acute care settings or case managers who may help older people without spouses or limited knowledge of the system navigate access to care. For younger older adults with a limited number of ADL limitations (short-delayed met needs), low-level training interventions may speed up the restoration of capacity for self-care (Gonzáles-Gonzáles & Requena, 2023). A subgroup of individuals with very high care needs seems to have been particularly vulnerable at the onset of needs as identified by one of the trajectories uncovered in this study. This group, together with older people without cohabitating partners, should merit particular attention when care needs are diagnosed or after acute health episodes. The prevalence of unmet needs long after the onset of needs (e.g., in the U-shaped met needs and long-delayed met needs) underscores the need for periodic assessments and screening of frail older people, for example, by general practitioners or nurses (Liimatta et al., 2016).

## Supplementary Material

igag042_Supplementary_Data

## Data Availability

SHARE data used in this manuscript are freely available from the SHARE website (https://share-eric.eu/) upon registration. Code used to assemble the dataset and for the analysis can be accessed from the authors upon reasonable request. This study was not preregistered.
