# Supplementary material for: A dynamic approach to unmet needs in long-term care for older people across Europe
Source: Innov Aging. 2026 Apr 29;10(8):igag042. doi: 10.1093/geroni/igag042 (PMC13329069; doi:10.1093/geroni/igag042)
Supplement: igag042_Supplementary_Data [file igag042_supplementary_data.pdf]

Innovation in Aging **Supplementary Material: Rodrigues et al. A dynamic approach to unmet needs in long-term care for older people across Europe.**

Supplementary Figure 1: Sample selection diagram

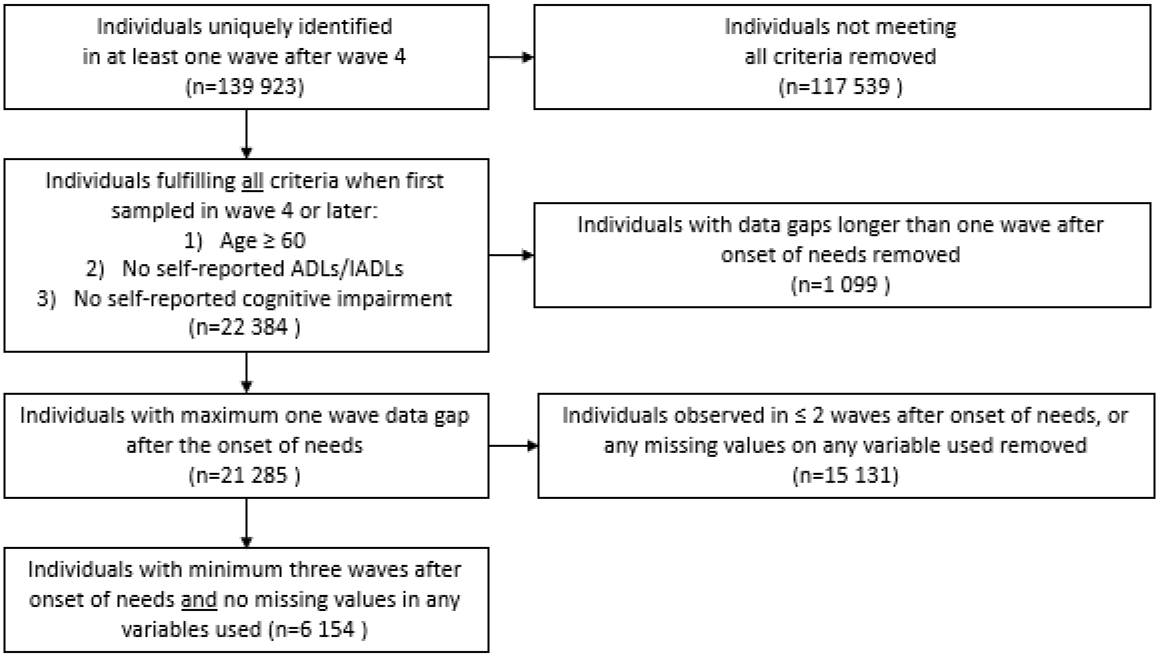

Supplementary Figure 2: State proportion plot for observations across waves

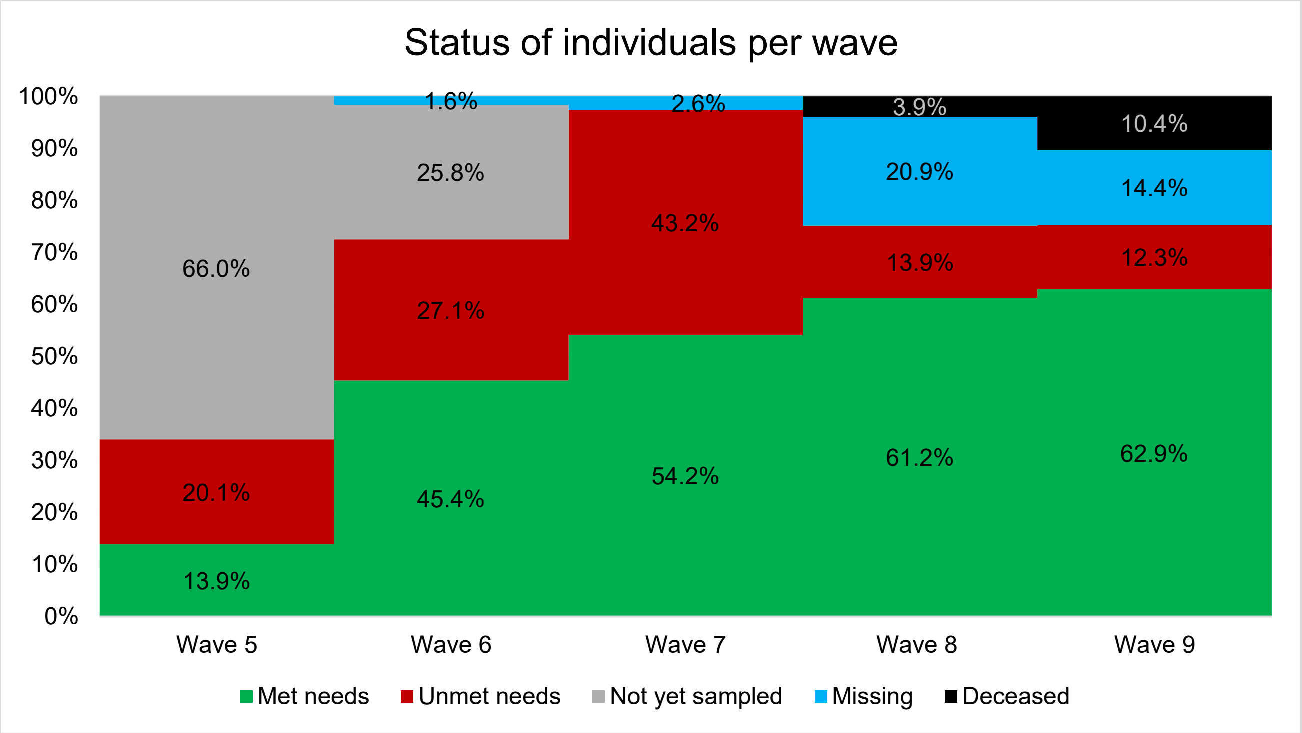

Source: Own calculations using SHARE waves 5-9.

Supplementary Figure 3: Trajectory membership and mean number of limitations with ADLs and IADLs across time

(A) Limitations with ADLs

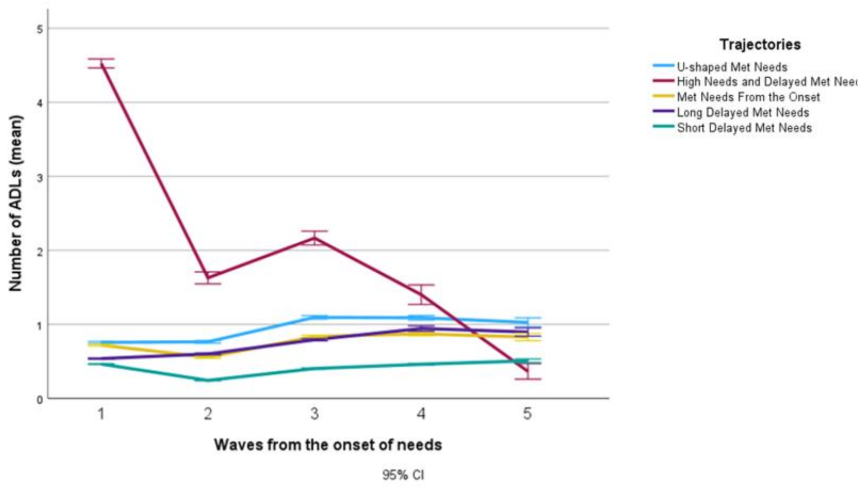

(B) Limitations with IADLs

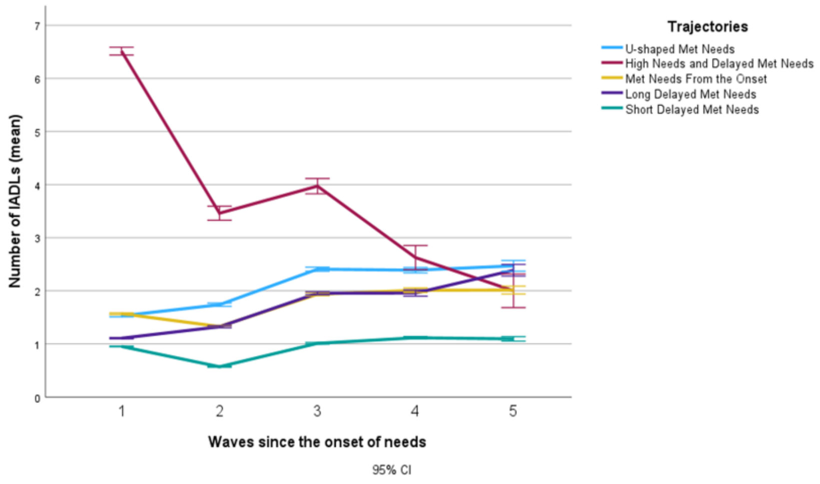

Notes: *N* = 6,154. 95% Confidence intervals.

Supplementary Table 1: Mean values of core characteristics by trajectory of unmet needs (weighted values)

Variables	U-shaped met needs	High needs and delayed met needs	Met needs from the onset	Long delayed met needs	Short delayed met needs
Time (waves) until first unmet need	1.53	0.37	4.99	0.07	0.003
Duration of unmet need (%)	31.55	43.26	0.10	74.67	33.04
No. changes between states	2.19	1.91	1.46	1.15	1.75
Age onset of needs	75.48	72.51	75.48	72.95	71.83
No. ADLs at onset	0.86	4.84	0.83	0.61	0.50
No of IADLs at onset	0.86	6.71	1.73	1.09	0.95
Share of total sample (%)	11.18	2.06	26.88	16.99	42.89

Notes. *N* = 6,154. ADL = activities of daily living; IADL = instrumental activities of daily living. Weighted results (calibrated cross-sectional individual weights for the first wave in which each individual joined the sample).

Supplementary Table 2: Multivariate analysis of determinants of trajectories of unmet needs (Average Marginal Effects) (weighted values)

Variables	U-shaped met needs		High needs and delayed met needs		Met needs from the onset		Long delayed met needs		Short delayed met needs	
	AME	*p*	AME	*p*	AME	*p*	AME	*p*	AME	*p*
Female (ref: male)	-0.009		-0.006	¥	0.042	***	-0.023	*	-0.003	
Partner in HH (ref: no partner in HH)	0.061	***	-0.005		0.160	***	-0.059	***	-0.156	***
Adult child in HH (ref: no adult child in HH)	-0.019		0.006		-0.056	**	0.039	*	0.030	
Income (ref: 1st quartile - poor)										
2nd quartile	0.058	***	0.003		0.070	***	-0.057	***	-0.075	***
3rd quartile	0.039	**	-0.0001		0.098	***	-0.057	***	-0.080	***
4th quartile	-0.006		-0.0004		0.120	***	-0.074	***	-0.040	¥
Education (ref: primary)										
Secondary	0.002		-0.005		-0.016		-0.047	***	0.066	***
Tertiary	-0.002		-0.007		0.022		-0.067	***	0.054	**
Country cluster (ref. Low expenditure)										
Mid-low expenditure	-0.034	*	0.006		0.043	**	-0.066	***	0.052	*
Mid-high expenditure	-0.030	*	-0.008		0.146	***	-0.108	***	0.001	
High expenditure	-0.078	***	-0.016	**	0.147	***	-0.105	***	0.052	*
Reduction in income (ref: no change or improvement)	-0.034	**	0.002		-0.016		-0.006		0.054	***
Reduction in HH size (ref: same size or larger)	0.008		-0.002		0.062	***	-0.051	***	-0.017	

Notes. *N* = 6,154. HH = household. Weighted results (calibrated cross-sectional individual weights for the first wave in which each individual joined the sample).
¥ p <0.1; * p < 0.05; ** p < 0.01; *** p < 0.001.
